# Consideration of underlying immunodeficiency in refractory or recalcitrant warts: A review of the literature

**DOI:** 10.1002/ski2.98

**Published:** 2022-02-09

**Authors:** J. Zampella, B. Cohen

**Affiliations:** ^1^ Ronald O. Perelman Department of Dermatology NYU Grossman School of Medicine New York New York USA; ^2^ Division of Pediatric Dermatology Johns Hopkins University School of Medicine Baltimore Maryland USA

## Abstract

Although the exact mechanisms have yet to be elucidated, it is clear that cellular immunity plays a role in clearance of human papillomavirus (HPV) infections as it relates to the development of warts. Patients with extensive, recalcitrant, or treatment‐refractory warts may have an underlying immune system impairment at the root of HPV susceptibility. Early recognition of genetic disorders associated with immunologic defects that allow for recalcitrant HPV infection may expedite appropriate treatment for patients. Early recognition is often pivotal in preventing subsequent morbidity and/or mortality that may arise from inborn errors of immunity, such as WHIM (Warts, Hypogammaglobulinemia, Infections, Myelokathexis) syndrome. Among these, cervical cancer is one of the most common malignancies associated with HPV, can be fatal if not treated early, and is seen more frequently in patients with underlying immune deficiencies. A review of diseases with susceptibility to HPV provides clues to understanding the pathophysiology of warts. We also present diagnostic guidance to facilitate the recognition of inborn errors of immunity in patients with extensive and/or recalcitrant HPV infections.

## Graphical abstract

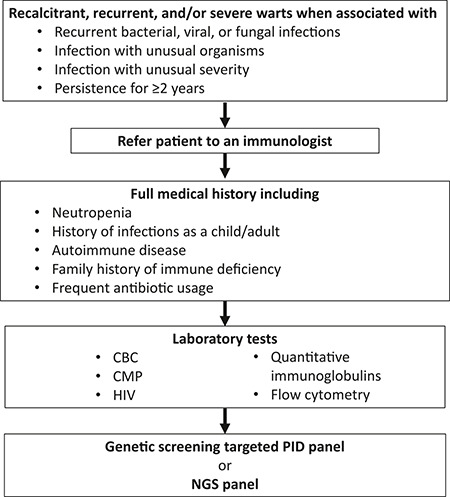

1

**What is already known about this topic?**

- Warts caused by human papillomavirus often resolve in healthy individuals with or without treatment; however, some patients develop persistent, treatment‐refractory warts.

**What does this study add?**

- Patients with recalcitrant warts may have an underlying immunodeficiency. Early diagnosis and treatment are important to limit progression to more serious morbidities, such as cancer. This review compiles information into one article to help specialists who may encounter patients with recalcitrant warts due to an underlying immunodeficiency.

## 2 INTRODUCTION

Human papillomavirus (HPV) is a ubiquitous and diverse species of viruses comprising >200 subtypes^1^, ^2^, ^3^ infecting cutaneous and mucosal tissues, leading to warts, papillomas and cancers.^1^, ^2^ In the United States, >40 million people are estimated to be infected with disease‐associated HPV, with 13 million having acquired a new infection in 2018,^4^ leading to high health care system burden ($8 billion in 2010).^5^ The prevalence and potential for significant morbidity and mortality from HPV may be higher in patients with underlying immunodeficiencies such as Warts, Hypogammaglobulinemia, Infections, Myelokathexis (WHIM) syndrome. Similarly, patients with undiagnosed inherited or acquired immunodeficiencies may demonstrate refractory or more severe HPV infections.^5^

## 3 AIMS AND METHODS

Herein, we aim to leverage the current literature and our clinical experience evaluating the underlying causes of HPV‐derived recurrent and recalcitrant warts. We conducted a PubMed search for articles containing the search terms ‘primary immunodeficiency’, ‘human papilloma virus (HPV)’ and ‘warts’. The search was performed in June 2021 and included English language results published at any time. Selection criteria for incorporation of the sources found in these searches consisted of relevance to the topic and primacy of the data. We then incorporated the published information on immunodeficiency in HPV infections and developed a proposed algorithm for evaluation and treatment of patients with severe or refractory HPV‐related infections.

## 4 HPV PATHOPHYSIOLOGY

HPV is divided into alpha, beta, gamma, mu and nu genera based on phylogenetic analysis.^3^ The genus gamma is largest (*n* = 99), followed by alpha (*n* = 65), beta (*n* = 54), mu (*n* = 3) and nu (*n* = 1).^6^ The majority of HPV research focuses on alpha and beta HPV, both of which are associated with cancer.^7^ Alpha HPV preferentially infects mucosal epithelia but has been associated with cutaneous warts in normal hosts; a small subset causes anogenital or head and neck cancer.^6^, ^7^ Beta HPV typically infects cutaneous epithelia such as nail beds and hair follicles, and particularly in combination with ultraviolet (UV) exposure, may lead to skin cancers.^7^ While less well understood, current conception of gamma HPV suggests that it rarely causes human disease, though some types may cause transient warts and, as emerging evidence indicates, may interact with tumour suppressor proteins such as p53.^3^ Mu and nu subtypes have been associated with warts.^6^

Unlike most viruses, multiple HPV strains may be present in a patient^3^, ^8^ but do not necessarily lead to disease,^9^ and the molecular mechanisms of disease are strain specific. Beta and gamma HPV rarely cause disease, but alpha HPV has immunoevasive strategies allowing viruses to persist and form papillomas.^10^ Entering a host cell, HPV hijacks host molecular machinery to replicate.^11^ Initially, the HPV genome is maintained in the basal epithelium by replicating alongside host cellular DNA. Upon differentiation of basal cells into the suprabasal layers, where host DNA replication machinery is suppressed, HPV‐encoded E6 and E7 proteins enable continued machinery utilisation and can delay epithelial cell differentiation. E6 and E7 combination further facilitates HPV replication by overriding cell cycle checkpoints.^11^ Ultimately, HPV speeds up cellular proliferation, induces blood vessel growth, and inhibits major histocompatibility complex expression.^12^ These processes induce the clinical features observed in cutaneous and mucosal warts, including hyperkeratosis, small dotted vessels, and disruption of normal skin architecture.^13^, ^14^ Each HPV strain induces different wart morphologies, leading to clinical manifestations of specific subtypes (Table 1).^13^, ^14^, ^15^, ^16^, ^17^, ^18^, ^19^, ^20^, ^21^

**TABLE 1:** Types of HPV warts and associated strains

Wart type	HPV strain
Common warts (includes filiform warts) (verruca vulgaris)^13^ ^,^ ^15^ ^,^ ^16^ ^,^ ^18^	1, 2, 3, 4, 7, 27, 29, 57, 75–77
Palmar and plantar warts (includes mosaic warts)^15^ ^,^ ^17^	1, 2, 4, 27, 57
Flat (plane) warts^13^ ^,^ ^15^	3, 10, 26–29, 41
Butcher's warts^13^	7
Cystic warts^13^	60
Genital warts^20^ ^,^ ^21^	6, 11, 16, 18, 26, 30, 31, 33, 34, 35, 39, 40, 42, 43, 44, 45, 51, 52, 53, 54, 56, 58, 59, 61, 62, 66, 67, 68, 69, 70, 71, 72, 73, 81, 82, 83, 84, 85, 89, 97
Ungual warts^19^	1, 2, 4, 27, 57
Oral warts^14^	6, 11, 32
Intermediate warts (features of common and flat warts)^14^	2, 3, 10, 28

*Note*: Various types of warts and the known strains of HPV that are associated with their appearance.

Abbreviation: HPV, human papillomavirus.

Perhaps the most consequential aspect of HPV physiology is its oncogenic potential. In women, high‐risk HPV is associated with cervical intraepithelial neoplasia (CIN), a precursor to invasive cervical cancer if not cleared by the host's immune system. In men, high‐risk HPV is associated with head and neck squamous cell carcinoma and penile cancer.^9^ Malignant transformation has been characterised primarily with alpha HPV, split into low and high risk based on propensity for malignancy.^7^, ^12^ The mechanisms underlying malignant transformation of alpha HPV stem from virally encoded E6 and E7 proteins that target apoptosis and cell cycle regulation via p53 and retinoblastoma tumour suppressor protein (pRb), respectively.^7^ E6 and E7 overexpression can lead to incorporation into the host genome, leading to cell immortalisation.^7^ Beta HPV is associated with cutaneous squamous cell carcinoma, but carcinogenesis mechanisms differ from high‐risk alpha HPV.^22^ While beta HPV likely shares oncogenic similarities with low‐risk alpha HPV, factors such as UV radiation, which can be locally immunosuppressive, may also be involved.^7^, ^23^, ^24^

## 5 HOST RESPONSE TO HPV

In immunocompetent hosts, HPV infection is eventually recognised and cleared. For subtypes causing genital infections, median HPV infection clearance is 5.9 months, regardless of whether low or high risk, and 75% of patients clear HPV infection within 12 months.^25^ HPV persists in the epithelium longer than most viruses, crucial for its infective process. Though mechanisms are being investigated, HPV clearly evades the immune system using several mechanisms, including blocking immune‐related gene expression, immune signalling pathways, and antigen presentation machinery that typically generates epitopes for T cells to identify infected cells.^26^ HPV enters through disruptions in the skin barrier—a first line of defence against HPV as evidenced by a higher risk of cutaneous HPV infections in children with atopic dermatitis.^11^, ^27^ Next, the virus binds via its L1 major capsid protein to heparan sulphate proteoglycans on the epithelial basement membrane and undergoes a change in conformation, exposing the N‐terminus of the L2 minor capsid protein. This prompts L2 proteolysis, which exposes a previously occluded surface of L1 that binds to an unknown receptor on keratinocytes that migrated to close the initial wound, allowing internalisation.^28^

Inability to clear HPV may predispose malignant transformation.^7^, ^12^ Cluster of differentiation 4 (CD4) and CD8 T cells are present in genital warts as they regress, along with adhesion molecule upregulation involved in lymphocyte recruitment. Lymphocytes produce cytokines such as interleukin‐12 (IL‐12), tumour necrosis factor‐alpha (TNFα), and interferon‐gamma (IFNɣ), which are characteristic of a T‐helper 1 (Th1) phenotype, a proinflammatory response leading to cellular immunity. Further evidence for CD4 T‐cell/Th1 response involvement in clearing HPV infections is that individuals with asymptomatic HPV infections have shown strong Th1 response to E2 and E6 HPV proteins, whereas patients with CIN show impaired Th1 response.^29^ Additionally, patients with cervical cancer due to HPV infection may have impaired CD8+ T‐cell activation, which leads to a lack of granzyme B and IL‐2R expression.^30^

In patients with immunodeficiencies, host recognition and clearance of HPV may be impaired and may perpetuate infection simultaneously with viral immune evasion strategies.^12^, ^29^ This combination may cause resistant, refractory, or severe warts; progressive papillomas; and eventual malignant transformation.^29^ In immunodeficient patients, non–disease‐causing strains may emerge, as evidenced by the recent discovery that beta and gamma HPV cause cutaneous warts.^3^, ^29^ Understanding dysfunctional immune system components in acquired and inherited immunodeficiencies can reveal immune pathways involved in HPV recognition, disease pathophysiology and host immune response.

## 6 IMMUNODEFICIENCIES AND HPV SUSCEPTIBILITY

Noninherited causes of immunodeficiency, including Human Immunodeficiency Virus (HIV), malignancy, connective tissue disease, chemotherapy, biologic therapy, and immunosuppressive agents, can lead to symptomatic HPV infections via impairment of several immune system components (Table 2).^2^, ^31^, ^32^, ^33^, ^34^, ^35^, ^36^ HIV leads to CD4+ helper T‐cell depletion, and women with HIV are more susceptible to HPV infection, particularly with low CD4+ cell counts.^35^ Similarly, altered immune function in diseases such as rheumatoid arthritis and systemic lupus erythematosus (SLE) is associated with increased HPV risk.^31^, ^36^, ^37^ SLE is characterised by complex underlying immune dysregulation, including amplification of Th‐type cytokines such as IFN‐γ, IL‐4, IL‐5, IL‐6, and TNF superfamily members mediating increased crosstalk between innate and adaptive immune responses, leading to autoreactive B‐cell activation and persistence.^38^ Cytokine milieu alterations may similarly predispose HPV persistence and immortalisation.^39^

**TABLE 2:** Causes of increased HPV susceptibility^2^, ^31^, ^32^, ^33^, ^34^, ^35^, ^36^

Acquired	Inherited
HIV	WHIM syndrome
Malignancy	Epidermodysplasia verruciformis
Connective tissue disease	DOCK8 deficiency
Chemotherapy	GATA2 deficiency
Biologic therapy	LAD1
Immunosuppressive agents	IL2RG/JAK3 deficiency
	Ataxia telangiectasia
	CD28 deficiency
	Netherton syndrome
	NEMO
	SCID
	Wiskott–Aldrich syndrome

*Note*: The causes of increased susceptibility to recurrent HPV infections, sorted by acquired and inherited causes. Many of the inherited causes are recognised inborn errors of immunity.

Abbreviations: DOCK8, dedicator of cytokinesis 8; GATA2, GATA binding factor 2; HIV, Human Immunodeficiency Virus; HPV, human papillomavirus; LAD1, leucocyte adhesion deficiency 1; NEMO, nuclear factor kappa‐B essential modulator; SCID, severe combined immunodeficiency; WHIM, warts, hypogammaglobulinemia, infections, and myelokathexis.

Many iatrogenic forms of immunosuppression increase HPV risk, including in patients receiving immunosuppression for solid organ transplants and patients receiving TNF inhibitors.^32^, ^36^ The increased risk of HPV attributable to TNF inhibitors may suggest a particular role for TNF in HPV clearance, evidenced by the capacity for TNFα to stimulate E6 and E7 proteins in HPV‐immortalised keratinocytes.^39^

Inherited syndromes and/or immunodeficiencies may also carry greater risk of HPV infections. Diseases such as epidermodysplasia verruciformis (EV), WHIM syndrome, leucocyte adhesion deficiency 1 (LAD1), hyper IgE syndromes caused by dedicator of cytokinesis 8 (*DOCK8*) mutations, GATA binding protein 2 (*GATA2)* mutations, interleukin 2 receptor subunit gamma (IL2RG) or Janus kinase 3 (JAK3) deficiency, ataxia telangiectasia (AT), and T‐cell CD28 deficiency can predispose patients to HPV infections in the form of warts and HPV‐related malignancies (Table 2).^29^, ^40^, ^41^, ^42^ Integrin subunit β2 (*ITGB2),* WASP actin nucleation promoting factor *(WAS),* adenosine deaminase 2 (*ADA2),* and NFKB inhibitor alpha (*NFKBIA)* also affect broad elements of the immune system and bring occasional susceptibility to HPV warts.^40^ Inherited immune defects found in the aforementioned conditions can provide additional insights into the immune response against HPV infection.

## 7 EPIDERMODYSPLASIA VERRUCIFORMIS

Epidermodysplasia verruciformis is caused by mutations in *EVER1* and *EVER2*, thought to encode zinc‐transport proteins.^29^, ^43^ Patients with EV are the archetype of genetic HPV susceptibility.^44^ The presence of decreased T lymphocytes and reduced cellular immunity (possibly due to impaired antigen presentation) underscores T‐cell function importance in HPV clearance.^29^ However, because patients with EV are not susceptible to other viral pathogens, there may be a specific role of *EVER1* and *EVER2* in keratinocyte immune response to HPV.^45^ In addition, patients with EV are prone to developing squamous cell carcinomas related to HPV infection, further highlighting the interplay between local (keratinocyte) and systemic immune response.^46^

Traditionally nonpathogenic strains of HPV (e.g., beta subtype) can cause disease in patients with EV.^29^ In EV, beta HPV susceptibility is hypothesised to result from a local TNF signalling defect,^47^ zinc transport dysfunction,^48^ or increased activity of transcription factors that override E5 protein absence in beta HPVs, which confers resistance in the presence of normal EVER protein function.^49^

## 8 WHIM SYNDROME

Individuals with WHIM syndrome are uniquely susceptible to cutaneous warts associated with gamma HPVs—which generally do not cause disease in immunocompetent individuals.^3^ These patients have a C‐X‐C chemokine receptor 4 (*CXCR4)* defect and experience recurrent bacterial infections due to neutropenia from impaired migration of polymorphonuclear cells to peripheral blood^50^, ^51^ and may be infected by multiple, simultaneous HPV strains.^3^ The broader risk of infection stemming from *CXCR4* mutations highlights the role of neutrophils in HPV immunity.^29^, ^52^, ^53^ Lymphopenias may also occur in WHIM syndrome, yet disproportionate susceptibility to HPV versus other lymphopenic disorders may suggest specific susceptibility in WHIM, namely the *CXCR4* gain‐of‐function mutation.^54^ *CXCR4* increases cell proliferation and immortalisation^55^ and increases TNFα expression^56^; both may contribute to HPV immunology.^11^, ^29^ Importantly, pharmacologic *CXCR4* antagonism reduced gamma HPV predominance in patients with WHIM syndrome over time in a clinical trial, a potential target for wart treatment.^3^

## 9 LEUCOCYTE ADHESION DEFICIENCY 1

LAD1 results in frequent skin and mucosal surface infection beginning in infancy and can be fatal.^57^ LAD1 is caused by genetic mutations in the common chain (CD18) of β2 integrin that profoundly impair leucocyte mobilisation to inflammation sites.^57^ Cutaneous and genital warts have been reported in patients with milder forms of LAD1; more severe forms lack this association due to bone marrow transplantation or death before manifestation.^29^

## 10 *DOCK8* MUTATIONS

Mutations underlying hyper IgE syndromes, such as *DOCK8*, present with a defect in dendritic cell and T‐cell migration, resulting in immunodeficiency characterised by increased cutaneous viral infection susceptibility, including HPV, herpes simplex virus, molluscum contagiosum, and varicella zoster virus.^29^, ^58^ IgE has an unclear role in HPV but could relate to changes in TNF‐α.^59^

## 11 *GATA2* MUTATIONS

Similar to *DOCK8*, missense or null mutations in *GATA2*, a transcription factor involved in haematopoiesis and stem cell maintenance, lead to a variety of presentations, with >75% of patients with *GATA2* deficiency being infected with HPV.^29^, ^60^ Importantly, *GATA2* mutations typically present in older children or even in adulthood^29^ with delayed diagnosis.^60^ *GATA2* haploinsufficiency can also be attributed to progressive multiple cytopenias, resulting in mycobacterial and fungal infections, and high risk of myelodysplastic syndrome, acute myeloid leukemia, lymphoedema, and pulmonary alveolar proteinosis. Around 50% of patients with *GATA2* haploinsufficiency have increased susceptibility to recurrent warts.^40^

## 12 *IL2RG* OR *JAK3* DEFICIENCY

Patients with *IL2RG* or *JAK3* deficiency have a reduced number of natural killer (NK) cells with decreased cytotoxic activity and, presumably, an impaired ability to eliminate virally infected cells.^42^ Patients with these deficiencies are known to carry a 50% risk of developing severe cutaneous HPV infections even after haematopoietic stem cell transplantation (HSCT), suggesting that the mutation either carries a defect intrinsic to the skin, or HSCT does not fully replace the faulty immunological component.^40^

One patient with a pathogenic germline X‐linked *IL2RG* mutation was shown to have a second, somatic mutation in *IL2RG* that reversed the T‐cell deficiency but left the NK cell population affected. The expansion of the HPV skin virome and recalcitrant HPV‐related skin and mucosal warts of this patient support a role for NK cells in HPV susceptibility and clearance. In this case, both benign and malignant HPV‐associated warts cleared with HSCT.^42^

## 13 ATAXIA TELANGIECTASIA

Ataxia telangiectasia (AT), which is caused by biallelic mutations of the *ATM* gene, affects multiple systems and leads to cerebellar degeneration, telangiectasia, immunodeficiency, and susceptibility to cancer. These mutations result in low B‐cell and naïve CD4+ and CD8+ T‐cell counts and abnormal B‐cell and T‐cell receptor repertoires due to the role of *ATM* in double‐stranded DNA breaks in V(D)J recombination.^40^ The *ATM* defect ultimately results in impaired antigen recognition, antibody production, and cytotoxic elimination of HPV‐infected cells, resulting in around 20% of patients developing persistent HPV warts.^40^, ^61^

## 14 INHERITED T‐CELL CD28 DEFICIENCY

A family with inherited hyperkeratotic cutaneous papillomatosis caused by HPV‐2 infection was identified to have a mutation in CD28. CD28, a costimulatory receptor expressed by T cells, is essential for interactions with antigen‐presenting cells. Patients carrying these mutations exhibit deficient CD4+ immune response against HPV, suggesting a dependence on HLA class II interactions for immunity against some HPV strains.^41^

The genetic pathologies listed above that are associated with susceptibility to HPV infection and warts all compromise essential elements of immune function, specifically viral immunity. Impaired T‐cell development, function, and/or mobilisation is present in *GATA2*, EV, and CD28 deficiency, while defects in antigen presentation are hallmarks of EV, CD28 deficiency, and *DOCK8* mutations.^40^, ^41^ Innate immune cells (e.g., NK cells and neutrophils), which play a role in elimination of virus infection response, are affected in LAD1 deficiency, *IL2RG/JAK3* deficiency, and WHIM syndrome.^42^, ^50^, ^51^, ^57^ All are essential to mounting an effective immune response to a viral pathogen; however, the very specific susceptibility to HPV and warts suggests a role for these immune elements in the HPV pathogenic mechanism itself.^41^

This highlights the importance of recognising potential signs of underlying immunodeficiency, which may include recalcitrant warts. Indeed, genetic mosaicism and incomplete gene penetrance in some immunodeficiencies may complicate diagnosis.^62^ Genetic testing has enabled earlier, more accurate diagnosis of suspected immunodeficiencies,^63^, ^64^ and LAD1 and WHIM syndrome can be confirmed through genetic testing despite heterogeneity in presentation.^50^, ^57^ Testing for these mutations can confirm diagnosis earlier and lead to a more targeted treatment approach.

## 15 CHARACTERISATION OF WART RECALCITRANCE, RECURRENCE, AND SEVERITY

With no universally accepted definition of treatment‐refractory, or recalcitrant, warts, a standard approach to evaluating and managing affected patients has been challenging. Leung suggested that warts are recalcitrant if not responding after five treatments over 6 months.^65^ This timeline may be too short, as warts often resolve with or without treatment within 18–24 months.^66^, ^67^, ^68^ Leung further estimated that approximately one‐third of common warts become recalcitrant^65^—a staggering number considering the overall prevalence of cutaneous warts. No currently accepted wart severity scale exists, which may be another important factor in understanding underlying immune phenotypes in patients with wart susceptibility. Recalcitrant and recurrent warts must also be distinguished. Recurrent warts resolve with treatment and reappear, suggesting underlying susceptibility to HPV infection as evidenced by HPV persistence and increased cervical cancer risk in certain human leucocyte antigen (HLA) subtypes.^65^, ^69^ We propose the definitions in Table 3. When observed, recalcitrant, recurrent, and/or severe warts should raise suspicion of underlying causes, specifically of inherited or acquired immunodeficiency.^29^, ^70^, ^71^

**TABLE 3:** Proposed definitions

	Definition
Recalcitrant warts	Warts that persist for >18 months despite consistent treatment with ≥2 accepted modalities
Recurrent warts	Wart recurrence within 3–6 months of clearance of prior warts on >2 different occasions
Wart severity	Based on the number, overall size, thickness and location of warts

*Note*: Proposed distinction between recurrent and recalcitrant warts as well as the factors influencing wart severity.

## 16 CONSIDERING IMMUNODEFICIENCY IN PATIENTS WITH RECALCITRANT WARTS

Prevalence of subclinical immune deficiencies is currently unknown. Nevertheless, in patients with severe, recurrent, or refractory warts, variations in aforementioned gene product expression and resulting immune deficiency may be considered possible components. Including immunodeficiencies in the differential diagnosis of recalcitrant warts may facilitate diagnosis in some patients. Considering current literature and direct experience in treating patients, we propose the following algorithm to aid clinicians in assessment and treatment of patients with recalcitrant warts in whom immunodeficiency should be suspected.

**FIGURE 1:**
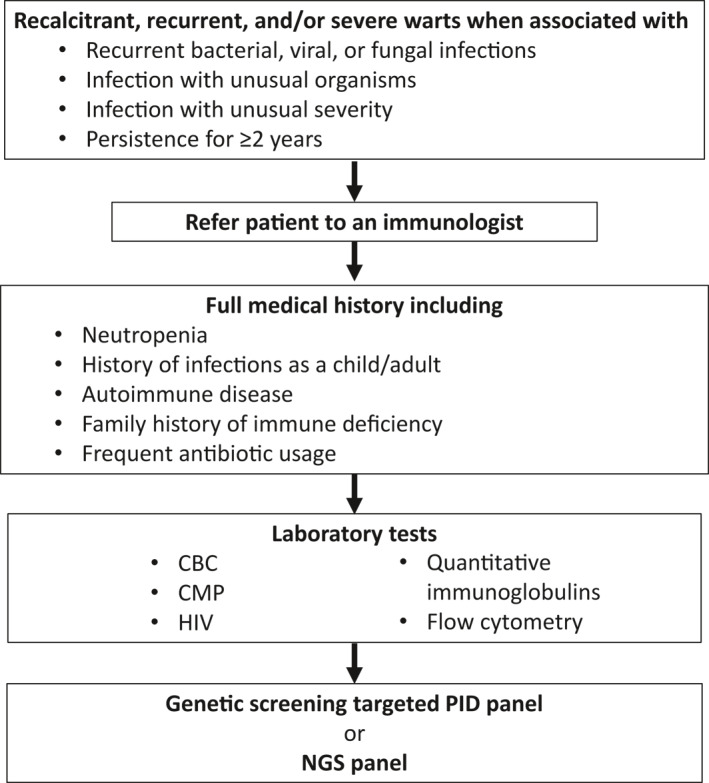
Diagnosis of underlying cause of recalcitrant warts. CBC, complete blood count; CMP, complete metabolic panel; NGS, next‐generation sequencing; PID, primary immunodeficiency

Patients with recalcitrant or recurrent severe warts should be evaluated for evidence of other infections (recurrent bacterial, viral, fungal or otherwise unusual or severe); this may be the first indicator of underlying immunodeficiency. History of recurrent infection, frequent antibiotic use since childhood, or autoimmune disease may raise suspicions of underlying immunity impairment. Additionally, family history of immunodeficiency suggests an inherited cause. With personal or family history suggestive of immunodeficiency or in warts persisting for >2 years, consideration of laboratory testing to exclude acquired and inherited forms of immune deficiency should be performed. Testing should include a complete blood count, comprehensive metabolic panel, HIV test, antinuclear antibody, and quantitative immunoglobulin assessments. If suspicion remains high and diagnosis elusive, genetic screening (first through a panel, and whole exome/genome sequencing if panel results are inconclusive) may confirm diagnosis (Figure 1).

## 17 TREATMENT OF HPV‐RELATED WARTS AND PAPILLOMAS

The HPV vaccine has led to substantial decrease in incidence and prevalence of HPV‐related cancers and genital warts; despite initial antibody increases, long‐term immunity is not always maintained in people with immunodeficiency.^72^, ^73^, ^74^, ^75^ Evidence is emerging on HPV vaccine treatment for common warts and other HPV‐related pathologies, suggesting cross immunogenicity between vaccine HPV subtypes and those causing cutaneous warts.^76^, ^77^

Many approaches exist to treat warts in healthy patients, although randomised controlled trials have mostly demonstrated equivalent efficacy, and no therapy will be effective in all patients.^78^, ^79^, ^80^ Common treatment approaches, which are summarised in Table 4, include destructive methods, such as salicylic acid or cryotherapy; chemotherapies, such as bleomycin; and immunotherapy, such as contact allergens, intralesional immunotherapy, imiquimod, or interferon.^7^, ^12^, ^81^, ^82^, ^83^ The significant risk of recurrence attached to both destructive and chemical interventions has resulted in the growing popularity of immunotherapy.^83^ Immunotherapy is a targeted approach used for treatment‐refractory warts and is aimed at stimulating the immune system to clear HPV.^83^ However, in patients with immunodeficiency, immunotherapies are often less effective.^29^ Imiquimod positively modulates the immune response by increasing cellular levels of multiple cytokines such as IFN‐α, IL‐6, and TNF‐α, with resulting antitumour and antiviral effects.^83^ Interferon has also shown promise in the treatment of HPV warts, due to its antiviral, antiproliferative, and proimmune effects. In a comprehensive review of 12 studies in patients infected with HPV receiving interferon, five of the studies demonstrated complete response rate in treating genital warts; however, no statistically significant difference was seen in recurrence. While the immune‐stimulatory effects are partially responsible for the success of interferon in otherwise healthy patients, its effectiveness in the treatment of warts in patients with inborn errors of immunity is less clear.^84^ Ustekinumab, an antibody against the p40 subunit of IL‐23 and IL‐12, blocks activity by inhibiting IL‐21–dependent production of IL‐17. This therapy was not evaluated for warts specifically, but a patient with LAD1 and intractable inflammatory lesions with dominant IL‐23 and IL‐17 signature saw improvement after 1 year receiving ustekinumab.^85^

**TABLE 4:** Common treatment approaches for warts

Therapy type	Examples	Comments
Destructive	Topical salicylic acid	Patient‐applied salicylic acid and physician‐applied cryotherapy (liquid nitrogen) are the most common treatments for cutaneous warts^81^
	Cryotherapy	
	Trichloroacetic acid	
	CO_2_ laser therapy	
	Excision^82^	
Immune modulating^81^, ^82^	Intralesional *candida* antigen	May be better for larger lesions
	Topical imiquimod	
	Th1‐stimulating vaccination^83^	
	Interferon	
Antiproliferative	Bleomycin	Target an underlying mechanism of HPV effect on the host genome^7^, ^12^
	Vitamin D analogues	
	Podophyllin	
	Podophyllotoxin	
	5‐fluorouracil^83^	
Antiviral	Cidofovir^83^	Retinoids offer the advantage of at‐home use^81^
	Retinoids^82^	

*Note*: Treatment strategies for warts encompass several categories of therapies based on their mechanisms of action. No single therapy is effective in every patient.

Abbreviations: CO_2_, carbon dioxide; HPV, human papilloma virus; Th1, T helper type 1.

In conclusion, HPV is a common human‐associated virus typically cleared by the host's immune system. In cases of unusual wart presentation (extensive, recalcitrant or treatment‐refractory), there may be underlying immunodeficiency warranting evaluation that may affect wart prognosis and management. Patients with suspected immunodeficiencies should be referred to an immunologist for evaluation, including genetic testing, as appropriate, to confirm or exclude inborn errors of immunity. For patients affected by such inborn errors, early diagnosis and tailored treatment could profoundly impact outcome, particularly in those patients who may go on to develop HPV‐related malignancies that can be fatal without prompt, targeted intervention.

## CONFLICT OF INTEREST

Zampella reports receiving consulting fees from X4 Pharmaceuticals. Cohen has no conflict of interest to declare.

## AUTHOR CONTRIBUTIONS

**John G Zampella:** Conceptualization, Data curation‐Supporting, Formal analysis, Funding acquisition‐Supporting, Investigation, Methodology‐Supporting, Project administration‐Supporting, Resources, Validation, Visualization, Writing ‐ original draft‐Supporting, Writing ‐ review & editing‐Supporting. **Bernard Cohen:** Conceptualization, Data curation‐Supporting, Formal analysis, Funding acquisition‐Supporting, Investigation, Methodology‐Supporting, Project administration‐Supporting, Resources, Validation, Visualization, Writing ‐ original draft‐Supporting, Writing ‐ review & editing‐Supporting.

## Data Availability

Data sharing not applicable to this article as no datasets were generated or analysed during the current study.
